# DaFiF: A complete dataset for fish's freshness problems

**DOI:** 10.1016/j.dib.2024.111016

**Published:** 2024-10-10

**Authors:** Eko Prasetyo, Nanik Suciati, Ni Putu Sutramiani, Adiananda Adiananda, Ayu Putu Wiweka Krisna Dewi

**Affiliations:** a Department of Informatics, Faculty of Engineering, Universitas Bhayangkara Surabaya, Jl. Ahmad Yani 114, Surabaya 60231, Indonesia; b Department of Informatics, Faculty of Intelligent Electrical and Informatics Technology, Institut Teknologi Sepuluh Nopember, Jl. Raya ITS, Surabaya 60111, Indonesia; c Department of Information Technology, Faculty of Engineering, Universitas Udayana, Jl. Raya Kampus Unud, Badung 80361, Bali, Indonesia; d Department of Electrical Engineering, Faculty of Engineering, Universitas Bhayangkara Surabaya, Jl. Ahmad Yani 114, Surabaya 60231, Indonesia; e Department of Aquatic Resources Management, Faculty of Marine Science and Fisheries, Universitas Udayana. Jl. Raya Kampus Unud, Badung 80361, Bali, Indonesia

**Keywords:** Fish, Freshness, Sensor Data, Images, Organoleptic

## Abstract

The fish are incorporated with ice to preserve their freshness when sold on the market. Ordinary people can only detect its freshness with some basic freshness knowledge. Therefore, non-destructive fish freshness inspection is an innovative solution to help. This dataset provides a medium to develop a system for non-destructive detection of fish freshness. There are three data variations: sensor data, images, and organoleptic examination. This dataset includes three fish species: mackerel, tilapia, and tuna, using 21 fish of each species. Data generation was carried out for 11 days, where 800 MQ (Metal Oxide) 135 and TGS (Taguchi Gas Sensor) 2602 sensor data and 80 images were generated every day. Organoleptic examinations were carried out using the Indonesian National Standard (SNI) 2729-2013 on six parameters: eyes, gills, body surface mucus, meat, smell, and body textures. This dataset can be used to develop a fish freshness detection system, regression modeling to estimate the deterioration in fish freshness, and standard grouping of freshness classes.

Specifications Table

**Table:** 

Subject	Computer Science, Agricultural Sciences
Specific subject area	Computer Vision and Pattern Recognition, Signal Processing, Aquaculture
Type of data	Raw, Chart, Image
Data collection	Three fish species were experimented with by storing them on ice for 11 days: mackerel, tilapia, and tuna. Daily, fish freshness is checked based on the odor emitted by the fish using a gas sensor and photographed using a cellphone camera. The result of the sensoring is the score of ammonia gas emitted by the fish. The inspection conducted on 19-29 January 2024 at 09.00-15.00. Every day, two testing sessions are carried out; each session is repeated two times, and each time, the test generates 20 sensor data and two images of each fish. At the same time, experts conducted organoleptic examinations on 11 other fish to confirm freshness.
Data source location	Data generation was carried out at the Laboratory of the Faculty of Marine Science and Fisheries, Universitas Udayana, Bali, Indonesia
Data accessibility	Repository name: Mendeley
	Data identification number: doi.org/10.17632/vx4ptwk3pb.1
	Direct URL to data: https://data.mendeley.com/datasets/vx4ptwk3pb/1
Related research article	This dataset is related to research on standardization of fish freshness classes with ice storage at https://doi.org/10.1016/j.ecoinf.2024.102533

## 1 Value of the Data

• This data represents the freshness value of fish stored on ice for 11 days using the MQ 135 and TGS 2602 sensors. The sensors read the gas emitted by the fish's body in a closed box. Data were generated on three fish species
• Numerical and image data are appropriate for modeling the downfall in fish freshness quality levels. Numerical data provides information on decreasing and increasing trends, while images provide appropriate work for freshness classification based on appearance. The organoleptic freshness score is a confirmation of freshness by sensors and cameras.
• Freshness quality is confirmed by experts using organoleptic methods and provide control sensors/cameras data.

## 2 Background

Fish is one of the daily diet menu of people because it is rich in nutrition [1] and easy to cook, as it contains protein, sodium, potassium, calories, and some fatty acid [2]. Therefore, fish freshness is the foremost important parameter when buying fish at the market [3,4]. Fish offered by sellers are generally stored with ice to retain their freshness and prevent deterioration [5]. However, the fish stored with ice continues to lose its freshness. Therefore, controlling the handling of newly harvested fish by retaining freshness should be a high priority [6]. The length of freshness level for four species of fish with ice is projected to be 6-22 days [7], but the latest study states the projection of fish with ice for up to 11 days using 6 organoleptic parameters [1]. Sensor score generated by sensors facilitate non-destructive inspection of fish freshness [8]. On the other hand, the camera also generates images to inspect the freshness of the fish without damaging it, either without or with extra tools [9]. Therefore, this dataset proposes data to build a non-destructive fish freshness inspection system using sensors and cameras. This dataset provides MQ (Metal Oxide) 135 and TGS (Taguchi Gas Sensor) 2602 sensor values on three fish species stored on ice for 11 days. Sensor data is confirmed by freshness checks by experts using organoleptic methods on six freshness parameters based on Indonesian National Standards (SNI). The images of the 11-day examination were also generated to detect the fish's freshness based on visual appearance, such as the head and tail [10], eyes [11], and whole body [12]. Comprehensive non-destructive fish freshness detection can be developed by combining sensor data, images, and organoleptic examination. The dataset will published for free after accepted in [13]. This dataset provided material requirements, i.e., datasets, to develop a non-destructive fish freshness detection system using data sensors and cameras. Development with machine learning, including classification, clustering, regression in analysis, and knowledge discovery, would improve community services, company management, and institutional development. Numerical and image data are appropriate for modeling the downfall in fish freshness quality levels. Numerical data provides information on decreasing and increasing trends, while images provide appropriate work for freshness classification based on appearance. The organoleptic freshness score is a confirmation of freshness by sensors and cameras.

## 3 Data Description

This dataset contains three interrelated pieces of information: sensor data, images, and organoleptic examination results. The data consists of checking fish freshness for 11 days. Previous studies conducted experiments to check fish freshness for 4 to 22 days [7]. The fish species, post-harvest product category, and cooling method influenced this period. Recent experiments on seven species, including Oreochromis Niloticus, Oreochromis Mossambicus, Eleutheronema Tetradactylum, stated that 11 days of freshness inspection was a sufficient duration to prove a decrease in the freshness of fish treated with ice storage [1]. Therefore, the fish were treated with ice storage for 11 days in our experiment. Every day, two examination sessions are carried out. The examination is carried out in 3 ways: sensors, cameras, and organoleptic. Inspection with sensors produces sensor data, cameras produce photos of fish being checked for freshness, and organoleptic inspection is used to confirm non-destructive inspection with sensors and cameras. Sensors and cameras are non-destructive examinations, while organoleptics are destructive freshness examinations. These two checks are combined to obtain a comprehensive non-destructive fish freshness dataset. During dataset generation, we used three species of fish: mackerel, tilapia, and tuna. We use ten samples for each species. If each sample generates 40 sensor data every session, two sessions every day, then in 11 days, we get approximately 800 data on each sensor. Each sensor data has a freshness class. To confirm organoleptic freshness, we have provided 11 fish other. The fish are examined every day one by one using 6 organoleptic parameters. This data is enough to provide health information for 11 days. For this reason, we only need 1 sample every day.

**Fig. 1:**
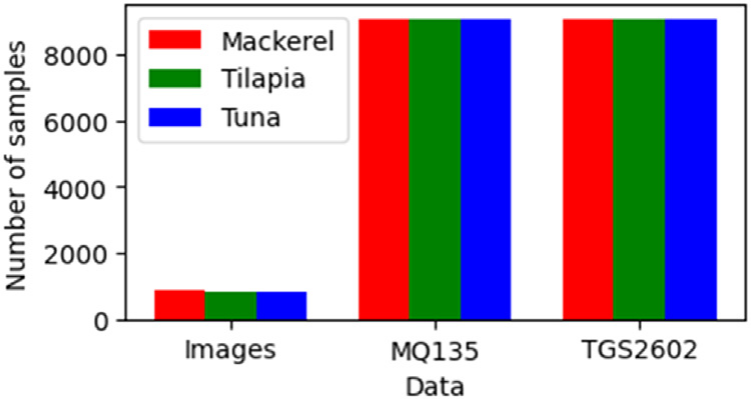
Samples image of fish.

**Fig. 2:**
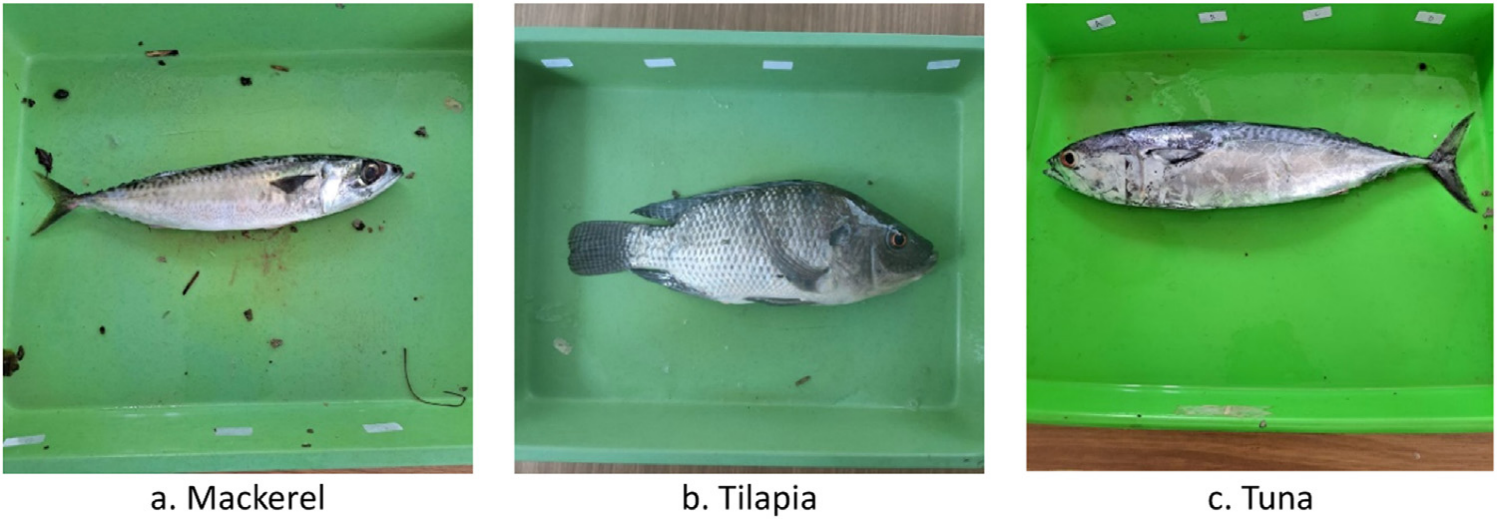
Samples image of fish

Non-destructive freshness inspections can be carried out visually by processing images produced by the camera [14]. For this reason, we photograph each fish at each inspection session to get a visual view of the fish's entire body. This method helps measure the level of freshness without destroying it based on the external appearance. The dataset is saved into 11 folders, each containing two subfolder sessions. Each session subfolder has sensor data and photographic images of 10 fish. We photographed each fish on two sides to obtain 40 images in each session. As presented in Table 1, on day 1 of session 2, the number of images obtained was 60, 40, and 60 for mackerel, tilapia, and tuna, respectively. The MQ 135 and TGS 2602 produce 421 sensor data for each fish species. Especially for day 1, there is only session 1, while there are both sessions on other days. The total images in the dataset are 859, 840, and 837, respectively, for mackerel, tilapia, and tuna. There are 9,041 sensors on both sensors and all fish species. The summary of the amount of data generated is presented in Fig. 1. We obtained 859, 840, and 837 in the image data for mackerel, tilapia, and tuna, respectively. This number is very satisfactory and balanced for all species. Sensor data all have the same amount, i.e., 9041 data. Sample fish images are presented in Fig. 2.

**Table 1:** Number of sample for images and sensor data.

**No.**	**Day**	**Sess.**	**Mackerel**			**Tilapia**			**Tuna**		
			**Images**	**Sensor data**		**Images**	**Sensor data**		**Images**	**Sensor data**	
				**MQ135**	**TGS2602**		**MQ135**	**TGS2602**		**MQ135**	**TGS2602**
1	1	2	60	621	621	40	621	621	60	621	621
2	2	1	40	421	421	40	421	421	40	421	421
3	2	2	40	421	421	40	421	421	40	421	421
4	3	1	40	421	421	40	421	421	40	421	421
5	3	2	40	421	421	40	421	421	20	421	421
6	4	1	40	421	421	40	421	421	40	421	421
7	4	2	40	421	421	40	421	421	40	421	421
8	5	1	40	421	421	40	421	421	40	421	421
9	5	2	40	421	421	40	421	421	40	421	421
10	6	1	40	421	421	40	421	421	40	421	421
11	6	2	40	421	421	40	421	421	40	421	421
12	7	1	39	421	421	40	421	421	41	421	421
13	7	2	40	421	421	40	421	421	40	421	421
14	8	1	40	421	421	40	421	421	40	421	421
15	8	2	40	421	421	40	421	421	40	421	421
16	9	1	40	421	421	40	421	421	40	421	421
17	9	2	40	421	421	40	421	421	40	421	421
18	10	1	40	421	421	40	421	421	36	421	421
19	10	2	40	421	421	40	421	421	40	421	421
20	11	1	40	421	421	40	421	421	40	421	421
21	11	2	40	421	421	40	421	421	40	421	421
Total			859	9,041	9,041	840	9,041	9,041	837	9,041	9,041

The organoleptic examination uses six parameters of the fish body according to the SNI 2729-2013 standards: eyes, gills, body surface mucus, meat, smell, and body textures. The freshness value scale determined by SNI is [1.9], where 9 represents the best freshness value while 1 represents the worst freshness.

SNI 2729-2013 guide assessing the freshness of fish by comparing the results of inspections by experts against standard tables. For example, for eyes, a value of 9 indicates convex eyeballs, clear, shiny corneas, and pupils specific to the type of fish. A fish eye with a value of 1 indicates that the eyeball is very sunken, the cornea is very cloudy, and the pupil is gray, not shiny. The generation of fish freshness data by sensors and cameras is confirmed by examination by experts using organoleptic methods simultaneously.

Organoleptic examination of fish freshness is carried out by examining the physical appearance of the fish and the odor emitted, including eyes (E), gills (G), body surface mucus (B), meat (M), smell (S) and body texture (T). Sensory results by experts are compared with Table 2 to provide a freshness score for each examination session. Organoleptic inspection is carried out simultaneously with the freshness inspection scenario using sensors and cameras. The organoleptic freshness inspection dataset is presented in Table 3.

**Table 2:** Criteria for fish freshness according to SNI 2729-2013.

**No.**	**Specification**	**Score**
**A.**	**Eyes**	
1.	The eyeballs are convex, the cornea and pupil are clear and shiny, specific to the fish species	9
2.	The eyeballs are flat, the corneas and pupils are clear, slightly shiny, specific to fish species	8
3.	The eyeball is flat, the cornea is slightly cloudy, the pupil is slightly grayish, slightly shiny, specific to fish species	7
4.	The eyeball is slightly sunken, the cornea is slightly cloudy, the pupil is slightly grayish, slightly shiny, specific to the fish species	6
5.	The eyeballs are slightly sunken, the cornea is cloudy. pupil slightly greyish, not shiny	5
6.	Sunken eyeballs, cloudy corneas, grayish pupils, not shiny	3
7.	The eyeballs are very sunken, the corneas are very cloudy, the pupils are gray, not shiny	1
**B.**	**Gills**	
1.	The gills are dark red or reddish brown, bright with very little transparent mucus	9
2.	The gill color is dark red or reddish brown, less brilliant with a little transparent mucus	8
3.	The gills are pink or light brown with a little mucus, slightly cloudy	7
4.	The gills are pink or light brown with slightly cloudy mucus	6
5.	The gills are pale pink or light brown with cloudy mucus	5
6.	The gills are gray or grayish brown with lumpy milky white mucus	3
7.	The color of the gills is gray, or grayish brown with lumpy brown mucus	1
**C.**	**Body surface mucus**	
1.	The mucus layer is clear, transparent, bright shiny	9
2.	The mucus layer is clear, transparent, quite bright	8
3.	The mucus layer begins to become slightly cloudy	7
4.	The mucus layer begins to become cloudy	6
5.	The mucus is quite thick, starting to change color	5
6.	Thick mucus slightly clots, color changes	3
7.	Thick mucus clots, changes color	1
**D.**	**Meat**	
1.	The meat incision is very bright, specific type, the meat tissue is very strong	9
2.	Type-specific brilliant meat incisions, strong meat tissue	8
3.	The cut of meat is a little less brilliant, the meat tissue is strong	7
4.	The meat incision is less brilliant; the meat tissue is less strong	6
5.	The meat incisions are starting to fade, the meat tissue is less strong	5
6.	The meat incision is dull, the meat tissue is less strong	3
7.	The meat incision is very dull, the meat tissue is damaged	1
**E.**	**Smell**	
1.	Very fresh, strong species specific	9
2.	Fresh, species specific	8
3.	Fresh, less species specific	7
4.	Neutral	6
5.	Slight sour smell	5
6.	More sour smell	3
7.	Strong sour smell	1
**F.**	**Body textures**	
1.	Dense, compact, very elastic	9
2.	Dense, compact, elastic	8
3.	A bit soft, a bit elastic	7
4.	A bit soft, a little less elastic	6
5.	A bit soft, less elastic	5
6.	Soft finger marks are visible and disappear very slowly	3
7.	Very soft, finger marks do not disappear	1

**Table 3:** The dataset of organoleptic freshness inspections.

**Day**	**Sess.**	**Mackerel**						**Tilapia**						**Tuna**					
		**E**	**G**	**B**	**M**	**S**	**T**	**E**	**G**	**B**	**M**	**S**	**T**	**E**	**G**	**B**	**M**	**S**	**T**
1	1	-	-	-	-	-	-	-	-	-	-	-	-	-	-	-	-	-	-
	2	8	9	9	9	9	8	9	9	9	9	9	9	9	9	9	9	9	9
	3	8	9	9	9	9	8	9	9	9	9	9	9	9	9	9	9	9	9
	4	8	9	9	9	9	8	9	9	9	9	9	9	9	9	9	9	9	9
2	1	8	9	9	9	9	7	9	9	9	8	9	9	8	9	9	9	9	9
	2	8	8	9	8	9	7	9	9	9	8	9	9	8	8	9	9	9	9
	3	8	8	9	8	8	7	9	9	9	8	8	9	8	8	9	8	9	8
	4	7	8	8	8	8	7	9	9	8	8	8	9	8	8	9	8	8	8
3	1	7	8	8	8	8	6	9	8	8	8	8	9	7	8	9	8	8	8
	2	7	8	8	8	8	6	8	8	8	7	8	8	7	8	9	8	8	7
	3	7	7	8	7	8	6	8	8	8	7	8	8	7	7	9	8	8	7
	4	7	7	8	7	8	6	8	8	8	7	8	8	7	7	8	8	8	7
4	1	6	7	8	7	8	6	8	7	7	6	7	8	7	7	8	7	8	7
	2	6	7	8	7	8	6	8	7	7	6	7	8	7	7	8	7	8	7
	3	6	7	7	7	7	6	8	7	7	6	7	8	7	7	8	7	8	7
	4	6	7	7	6	7	6	7	7	7	6	7	8	7	7	8	7	7	6
5	1	6	6	7	6	7	5	7	6	7	6	7	7	6	6	7	7	7	6
	2	6	6	7	6	7	5	7	6	7	6	7	7	6	6	7	6	7	6
	3	6	6	7	6	7	5	7	6	7	6	7	7	6	6	7	6	7	6
	4	6	6	7	6	7	5	7	6	7	5	7	7	6	6	7	6	7	6
6	1	6	6	6	6	7	5	6	6	7	5	7	7	5	6	7	6	7	5
	2	6	6	6	6	7	5	6	6	7	5	7	7	5	6	7	6	7	5
	3	5	6	6	6	7	5	6	6	7	5	7	7	5	6	7	6	7	5
	4	5	5	6	6	7	5	6	6	6	5	7	6	5	5	7	6	7	5
7	1	5	5	6	6	6	5	6	6	6	6	7	6	5	5	7	6	7	5
	2	5	5	6	5	6	5	5	6	6	6	7	6	5	5	6	5	7	5
	3	5	5	6	5	6	5	5	6	6	6	7	6	5	5	6	5	7	5
	4	5	5	6	5	6	5	5	6	6	6	7	6	5	5	6	5	7	5
8	1	3	5	5	5	6	5	5	5	6	6	6	6	5	5	6	5	7	5
	2	3	5	5	5	6	3	5	5	6	6	6	6	5	5	6	5	7	5
	3	3	5	5	5	6	3	5	5	6	6	6	6	5	5	6	5	7	5
	4	3	5	5	5	6	3	5	5	6	6	6	6	5	5	6	5	7	5
9	1	3	3	5	5	6	3	3	5	6	5	6	5	3	3	6	3	6	3
	2	3	3	5	3	5	3	3	5	6	5	6	5	3	3	6	3	6	3
	3	3	3	5	3	5	3	3	5	6	5	6	5	3	3	6	3	6	3
	4	3	3	5	3	5	3	3	5	6	5	6	5	3	3	6	3	6	3
10	1	1	3	3	3	5	1	3	3	6	3	0	5	3	3	6	1	5	3
	2	1	3	3	1	5	1	3	3	6	3	6	5	3	3	6	1	5	3
	3	1	3	3	1	5	1	3	3	6	3	5	5	3	3	6	1	5	3
	4	1	3	3	1	5	1	3	3	6	3	5	5	3	3	6	1	5	3
11	1	1	1	3	1	5	1	3	3	5	3	5	5	1	1	5	1	5	1
	2	1	1	3	1	5	1	3	3	5	3	5	5	1	1	5	1	5	1
	3	1	1	3	1	5	1	3	3	5	3	5	5	1	1	5	1	5	1
	4	1	1	3	1	5	1	3	3	5	3	5	5	1	1	5	1	5	1

## 4 Experimental Design, Materials and Methods

Dataset generation was carried out using several Indonesian National Standard (SNI) literature and preparations. Fish and auxiliary materials used during freshness inspection are presented in Table 4. Selection of fish as raw materials, auxiliary materials, fish handling methods, organoleptic inspection methods, and assessment refer to SNI as follows:

1. SNI 2729:2013 about fresh fish
2. SNI 01-2729.1-2006 about fresh fish - specifications
3. SNI 01-2729.2-2006 about fresh fish - requirements and raw materials
4. SNI 01-2729.3-2006 about fresh fish - handling and processing
5. SNI 01-2346-2006 about organoleptic and/or sensory testing instructions
6. SNI 01-4872.1-2006 about ice for fish handling – specifications

**Table 4:** Fish and auxiliary ingredients for freshness inspection.

**No.**	**Materials**	**Explanation**
1.	Fresh fish	• Species: mackerel, tilapia, tuna Average weight of fish: mackerel 350 grams, tilapia 400 grams, tuna 300 grams • About 4-6 hours after harvest • The number of fish per type is 10 for freshness testing with sensors and 11 for organoleptic testing
2.	Ice	• Ice *tube*. • The ratio of the fish and ice used is 1:1
3.	Styrofoam	• Box for storing fish and ice • Each fish species is stored in 1 styrofoam
4.	Masking tape	Media for sealing Styrofoam covers
5.	Knife	Media to open the fish body during freshness inspection
6.	Gloves	Media to protect hands and fish hygiene during organoleptic examination

Fish freshness inspection, at the Laboratory of the Faculty of Marine Sciences and Fisheries, Universitas Udayana, Bali, Indonesia, uses the following procedures:

### 4.1 Preparation for storing fish

There were 11 new fish samples (around 4-6 hours after harvest), and one fish was set aside to inspect its freshness on the first day. The rest (10 fish) were put in a styrofoam box with ice in a ratio of 1:1 (1 kg of fish to 1 kg of tube ice). We use a ratio of 1:1 for ice and fish in accordance with general standards, where 1:1 ice and fish provide the ability to preserve freshness at a temperature of 0-2 degrees Celsius [1]. The fish are arranged in the box in tiers with ice, meaning the first layer of ice, the second layer of fish, the third layer of ice, the fourth layer of fish, and so on until all the fish can fit into the box. To inspect freshness using sensors and cameras, we also prepare ten fish samples with the exact handling.

### 4.2 Daily freshness examination

Every day from 09.00-15.00, the Styrofoam box is opened. We discarded the defrosting water, then picked one fish for examination. The remaining fish were again stored in a Styrofoam box with ice in a 1:1 ratio. Fish inspection is carried out based on 6 fish freshness parameters according to SNI 2729-2013 standards: eyes, gills, body surface mucus, meat, smell, and texture. The examination is carried out by observing the eyes, removing the gills cover to observe the gills, touching the surface of the skin to determine the body's mucus, opening the body to observe the meat, smelling the fish's body, and feeling the surface of the meat to observe the texture of the meat. The examination was carried out in 2 sessions; each session was carried out two times. On other hand, ten other fish were also checked for freshness using sensors and cameras. The fish were put into a Styrofoam box one by one and then closed. Next, the application running on the computer carries out detection to obtain 20 sensor data. After that, it is replaced with the next fish. This session also involved photographing fish with a camera; the resolution used was 9mp. We use ten samples per session for inspection by the sensor. This inspection is the primary inspection in accordance with the concept of non-destructive freshness inspection. It means that 10 fish were taken from the styrofoam to be examined and then returned to the styrofoam again. Meanwhile, organoleptic examination uses one sample every day. This sample is not the ten samples used previously but is stored in the same Styrofoam.

**Fig. 3:**
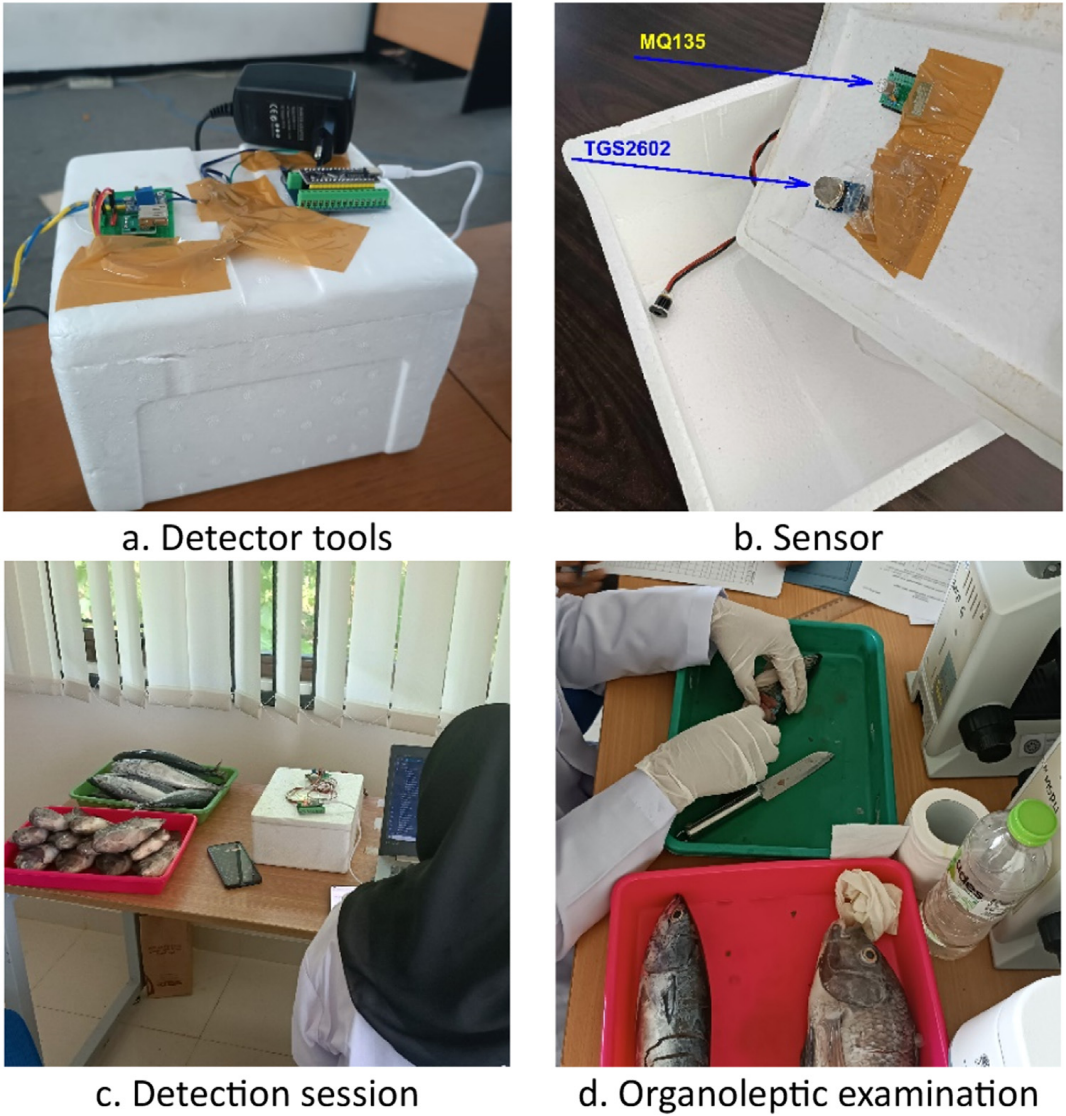
Data acquisition stage.

A prototype electronic assembly is a tool used to detect fish freshness with a sensor. The two sensors have been assembled into a detection device installed in a Styrofoam box and connected to a computer. Then, one by one, each fish was put into a Styrofoam box for freshness detection. The acquisition process is presented in Fig. 3.

The fish freshness level sensor provides the value of ammonia gas captured by the MQ 135 and TGS 2602 sensors in a closed room. This tool is also equipped with a camera placed in a box measuring 20 cm x 20 cm x 20 cm. Therefore, three varieties of data are generated: sensor data, images, and organoleptic examination. The data sensor uses 10 fish samples, which generate data in 2 sessions. Each session consisted of 2 detections, where 20 rows of sensor data of each type were generated for each detection. Consequently, in 1 session, 400 fish data were obtained, and every day, 800 sensor data were obtained; except for the first day, only one session was carried out with three detections, so 600 data were obtained. The final number of confirmed sensor data is in Table 1. The organoleptic examination used 11 fish, where one fish was used every day to check its freshness using the organoleptic method. Fish used in organoleptic examinations are not returned.

We acquired the fish as an image at each sample examination using a camera with specifications: Samsung model SM-A226B with ISO speed ISO-626 without flash. The image's dimensions are square, with a size of 3000×3000 pixels and a resolution of 96 dpi horizontally and vertically, with 24 bits in depth. Acquisition was carried out by hand-held at a distance of 20-30 centimeters with an inclination of 0-10 degrees from the vertical axis.

## Limitations

This dataset provided material requirements, i.e., datasets, to develop a non-destructive fish freshness classification system using data sensors and cameras. We get sensor and image data as the primary data. Its use is limited to classification, clustering, and regression systems using machine learning, deep learning, or others. Applications need to be embedded on mobile devices, such as the Internet of Things, using small devices such as Raspberry and Arduino to be applied real and mobile.

## Ethics Statement

This experiments complied with the ARRIVE guidelines and were carried out in accordance with the U.K. Animals (Scientific Procedures) Act, 1986 and associated guidelines; EU Directive 2010/63/EU for animal experiments; or the National Institutes of Health guide for the care and use of laboratory animals (NIH Publications No. 8023, revised 1978).

## CRediT authorship contribution statement

**Eko Prasetyo:** Conceptualization, Methodology, Investigation, Funding acquisition, Formal analysis, Project administration, Data curation, Validation, Visualization, Writing – original draft. **Nanik Suciati:** Investigation, Formal analysis, Supervision, Writing – review & editing. **Ni Putu Sutramiani:** Investigation, Formal analysis, Writing – review & editing. **Adiananda Adiananda:** Software, Data curation, Validation, Visualization, Resources. **Ayu Putu Wiweka Krisna Dewi:** Data curation, Validation, Visualization.

## Data Availability

Mendeley DataDataset for Fish's Freshness Problems (Original data).
